# Reversible stress cardiomyopathy in Guillain-Barré syndrome: a case report

**DOI:** 10.1186/s13256-019-2085-9

**Published:** 2019-05-20

**Authors:** A. Gravos, A. Destounis, K. Katsifa, P. Tselioti, K. Sakellaridis, V. Grammatikopoulou, C. Tsapas, A. Nodarou, P. Batiani, A. Prekates

**Affiliations:** Intensive Care Unit (ICU), Tzaneio General Hospital of Piraeus, Dodonis 26, 13451 Kamatero, PC Greece

**Keywords:** Guillain-Barré syndrome, Cardiomyopathy

## Abstract

**Background:**

Guillain-Barré syndrome is an autoimmune disorder in which autoantibodies mainly affect the peripheral nervous system. Autonomic dysfunction is a common and severe complication of Guillain-Barré syndrome. Cardiomyopathy, though, is a rare complication in Guillain-Barré syndrome, with only a few cases reported in the literature.

**Case Presentation:**

We present a case of a 65-year-old Greek woman with Guillain-Barré syndrome who developed cardiomyopathy shortly after admission to the intensive care unit due to respiratory deterioration. Her estimated left ventricular ejection fraction upon admission was 20%. The result of coronary angiography was negative for coronary artery disease, and cardiac magnetic resonance imaging excluded myocarditis. Her clinical condition improved with supportive therapy, and her estimated left ventricular ejection fraction at discharge was normal.

**Conclusions:**

Clinicians should be aware of this potentially lethal complication of Guillain-Barré syndrome and the therapeutic options, because early diagnosis can improve prognosis. Routine electrocardiographic and echocardiographic assessments should be performed in patients with Guillain-Barré syndrome presenting with hemodynamic instability.

## Introduction

Guillain-Barré syndrome (GBS) is an autoimmune disease mainly affecting the peripheral nervous system. It may present with autonomic dysfunction (hypotension, hypertension, sinus tachycardia, paroxysmal tachyarrhythmias or bradyarrhythmias, and electrocardiographic [ECG] changes). Manifestations of GBS vary from monoparesis to life-threatening paralysis of the respiratory muscles [1–4].

Cardiovascular abnormalities in GBS are attributed to autonomic neuropathy and are seen variably in two-thirds of affected patients [2]. There have been few case reports associating GBS and ECG abnormalities or left ventricular dysfunction [5–12]. Usually explained by temporary alterations in cardiac innervations or catecholamine cardiotoxicity, ECG abnormalities are often regressive.

The pathophysiology remains unclear, but the role of catecholamine-mediated myocardial stunning may be predominant. The association of GBS with stress cardiomyopathy is not well understood. Dysregulation of autonomic tone with excessive sympathetic activation in GBS with elevated catecholamine levels has been reported. The dysregulation of the parasympathetic and sympathetic systems is responsible for alterations in peripheral vascular resistance, most often causing transient or permanent hypotension [13].

Rare cases of sudden cardiac death or cardiovascular collapse might be attributed to lethal arrhythmias or acute heart failure episodes, which could be prevented by transthoracic echocardiographic (TTE) examination and hemodynamic continuous monitoring [4, 8].

## Case presentation

### Patient information

We present a case of a 65 year-old Greek woman who presented to the neurology ward of our hospital with a 1-week history of symmetrical weakness of her lower limbs, numbness and paresthesia of her upper limbs, and dysarthria. Her medical, family, and psychosocial histories were unremarkable. She was not receiving any medication at the time of her presentation, and she had no allergies. She only reported an upper respiratory viral infection 2 weeks ago.

### Clinical findings

On neurological examination, the patient’s motor strength was 4/5 in her upper extremities and 1/5 in her lower extremities. The tendon reflexes were absent, and there was no cranial nerve involvement. Initially, there were no associated cardiac symptoms, no neuromuscular respiratory weakness (vital capacity [VC] > 20 ml//kg and maximal inspiratory pressure [MIP] > 30 cm H_2_O), and no hypercapnia (partial pressure of carbon dioxide [PCO_2_] = 38 mmHg) in arterial blood gas analysis. The patient was afebrile (36.8 °C), had normal ECG findings (sinus rhythm ~ 80 beats/min), and was hemodynamically stable (mean arterial pressure [MAP] = 70 mmHg). Initial cerebral magnetic resonance imaging (MRI) findings were normal. Both neurophysiological and cerebrospinal fluid (CSF) examinations were consistent with the diagnosis of GBS. Thus, CSF examination showed elevated protein level (450 mg/L) with normal cells (2/mm^3^), and electrodiagnostic testing showed temporal dispersion, significantly slow conduction velocities, prolonged distal and F-wave latencies, and abnormal upper extremity sensory nerve conduction. The patient’s laboratory test results upon admission were normal. Treatment with intravenous immunoglobulin on day 0 over a 5-day period (400 mg/kg/day) was started.

One day after admission to the neurology ward, intubation was necessary because of progressive respiratory failure (VC < 15 ml/kg and MIP < 20 cm H_2_O, PCO_2_ = 60 mmHg, pH = 7.24) due to muscle weakness and mucus plugging, and the patient was transferred to the intensive care unit (ICU). Shortly after an uncomplicated intubation (for which she received midazolam 10 mg and propofol 150 mg, without myochalasis), a marked increase in heart rate (sinus rhythm ~ 150 beats/min) was noted, and the patient became hemodynamically unstable (MAP = 50 mmHg), despite fluid loading.

### Diagnostic assessment

**Fig. 1:**
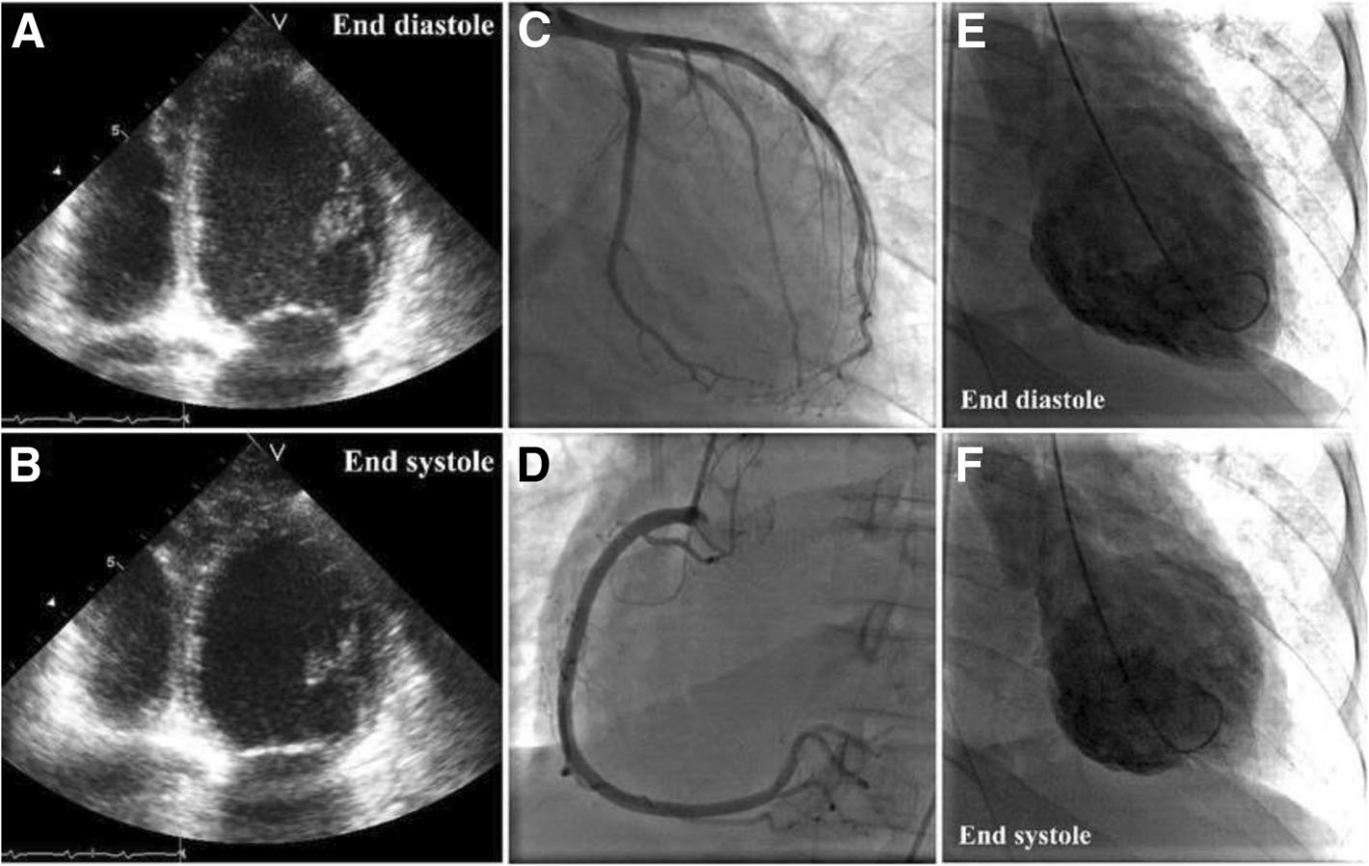
Echocardiographic four-chamber view (**a**, **b**), coronary angiography (**c**, **d**), and ventriculography (**e**, **f**) of the patient during the acute phase

**Fig. 2:**
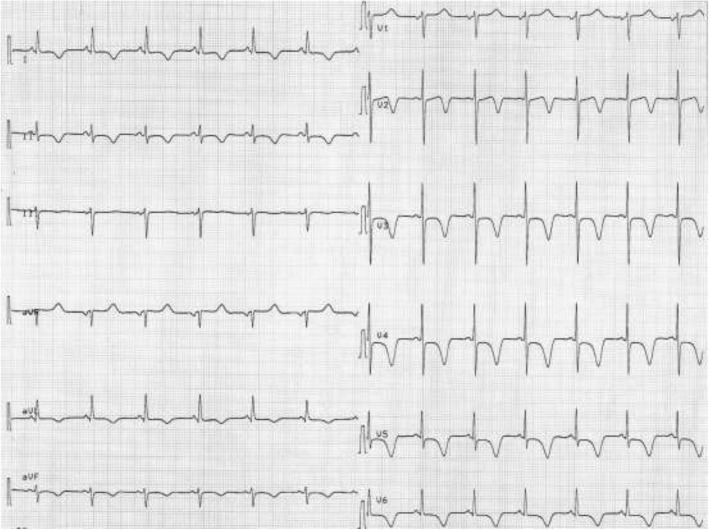
Electrocardiogram of the patient during hemodynamic instability

To rule out pulmonary embolism, computed tomography (CT) was performed, which only revealed atelectasis of the left lower lobe and no signs of pulmonary embolism. In the following hours, antibiotics, additional fluids, high-dose norepinephrine (80 μg/min), and hydrocortisone were administered. The patient’s MAP remained low (60 mmHg), tachycardia persisted (sinus rhythm ~ 120 beats/min), and urine output ceased. ECG revealed sinus tachycardia with nonspecific ST-T segment changes. Blood culture results and control for viral infections were negative. Laboratory tests revealed normal white blood cells; normal platelets and hematocrit; normal liver, thyroid, and kidney function; normal creatine kinase (CK = 56 U/L, normal < 145 U/L), but elevated troponin I (598 ng/L, normal < 14 ng/L) and N-terminal pro-brain natriuretic peptide (1391 pmol/L, normal < 15 pmol/L). Urgent TTE was performed, which revealed dilated and severe hypokinetic left ventricle, normal heart valves, normal right ventricle, and lack of pericardial effusion (Fig. 1a, b). The estimated left ventricular ejection fraction (LVEF) was 20%. A new ECG was performed, which showed inverted T-waves in leads I, avL, and V_2_–V_6_ (Fig. 2). Urgent coronary angiography to exclude coronary artery disease was performed, which was normal (Fig. 1c, d), but ventriculography revealed severe diffuse hypokinesis of the left ventricle (Fig. 1e, f). To exclude myocarditis, the patient underwent cardiac MRI on the tenth day of admission, which showed no signs of ischemia, fibrosis, or edema.

### Therapeutic interventions

On the basis of echocardiographic findings, it was concluded that the patient had stress cardiomyopathy or fulminant myocarditis. Dobutamine infusion was initiated (15 μg/kg/min) to assist left ventricular contractility, to reduce norepinephrine infusion, and to reduce afterload, despite persistent tachycardia. This resulted in a gradual increase in blood pressure and return of diuresis. The next day, additional furosemide was given because of a positive fluid balance and signs of pulmonary congestion on a chest x-ray. In the next 48 h, dobutamine was tapered because the patient gradually became hemodynamically stable. Due to persistent sinus tachycardia, metoprolol was introduced stepwise over the next 3 days to reduce the sympathetic tone and improve myocardial work/oxygen consumption ratio; the maintenance dose was 50 mg twice daily. After hemodynamic stabilization, a low dose of ramipril (2.5 mg/day) was also introduced. The diagnosis of myocarditis was excluded by cardiac MRI on the tenth day of admission.

### Follow-up and outcomes

Repeat follow-up TTE showed gradual normalization of LVEF in the next few days. Because of difficult weaning, the patient underwent tracheostomy on day 15, and she was discharged from the ICU on day 28 on spontaneous breathing. ECG and LVEF at discharge were normal, but the patient still had heavy peripheral, symmetrical, and especially motor polyneuropathy. New neurophysiological testing was not performed.

## Discussion

We present a case of a woman diagnosed with GBS who developed reversible stress cardiomyopathy. Massive catecholamine release due to the stressful event of rapid respiratory deterioration and hemodynamic instability after induction of anesthesia most likely caused the development of cardiomyopathy. In addition, norepinephrine infusion could have aggravated catecholamine excess, which might have contributed to the myocardial dysfunction. Takotsubo cardiomyopathy has been described in patients with GBS [5–12]; however, our patient presented with a type of stress cardiomyopathy (severe diffuse hypokinesis of the left ventricle) without the classical characteristics of takotsubo cardiomyopathy (transient hypokinesis, akinesis, or dyskinesis in the middle/apical segments of the left ventricular wall, with basal hyperkinesis).

Stress cardiomyopathy can be difficult to distinguish from the more common cardiovascular complications in GBS, owing to autonomic dysfunction such as tachyarrhythmias and bradyarrhythmias, blood pressure fluctuations, acute coronary syndromes, and myocarditis [4]. Coronary angiography and cardiac MRI are needed in order to exclude coronary artery disease and myocarditis.

The name “takotsubo” refers to a Japanese jar used by fishermen to catch octopuses. The round bottom but tight neck resemble a picture often seen by echocardiography of the left ventricular wall, called “apical ballooning” [14]. As more reports were published, it became clear that wall movement disorders were not restricted to the apex but could involve multiple segments of the left ventricular wall, as in our patient [15, 16].

The exact pathogenic mechanism of takotsubo cardiomyopathy is still controversial. The catecholamine hypothesis, which is that takotsubo cardiomyopathy is commonly induced by physical and/or emotional stress, seems the best explanation [16]. Sympathetic excitation of the brain triggers the release of the catecholamines norepinephrine and epinephrine, resulting in hyperdynamic basal contraction and apical systolic dysfunction. Takotsubo cardiomyopathy is a specific type of a broad spectrum of reversible cardiomyopathies, often stress-related [14, 17, 18]. In subarachnoid hemorrhage, pheochromocytoma, traumatic brain injury, and other neurological emergencies, excessive catecholamine release secondary to the primary insult has been reported, causing what is known as neurogenic stress cardiomyopathy/neurogenic stunned myocardium [19–24].

Transient cardiac dysfunction in GBS could also be attributed to aberrant immune responses directed against myocardial cells and peripheral nerves [8]. Alternatively, abnormal myocardial blood flow due to sympathetically mediated microvascular dysfunction has been suggested and is supported by decreased coronary flow reserve during the acute phase [25]. Furthermore, myocardial necrosis could result from increased sympathetic tone, and catecholamines could be the pathologic substrate of this transient cardiac dysfunction [25]. This is supported by the observation that plasma catecholamine levels are markedly elevated in acute stress cardiomyopathy compared with acute myocardial infarction [25]. An alternative explanation is the direct effect of catecholamines on cardiac myocytes. High levels of circulating epinephrine trigger a switch in intracellular signal trafficking. This change in signaling might be negatively inotropic. Despite these reports, a causal link between catecholamine exposure and stress cardiomyopathy has not been convincingly demonstrated [18].

On the basis of this background, the use of β-receptor-stimulating agents in patients with stress cardiomyopathy and GBS who have severe hypotension seems inappropriate. Non-β-receptor-stimulating agents such as vasopressin or terlipressin might be beneficial [26, 27].

Few cases associating GBS with reversible stress cardiomyopathy have been reported in the literature. In most of them, cardiomyopathy presented within the first week of hospital admission and completely resolved on discharge, after days or weeks [5–12]. Complications include heart failure (17.7%), recurrence (3.5%), and mortality (2.7%) [28]. Treatment consists of angiotensin-converting enzyme inhibitors, β-blockers, and diuretics in hemodynamically stable patients. β-blockers are believed to reduce sympathetic tone and improve the myocardial work/oxygen consumption ratio [28]. In hemodynamically unstable patients, the use of norepinephrine may be counterproductive, and treatment must be individualized for each patient. Treatments used in this setting include enoximone, a phosphodiesterase inhibitor that has positive inotropic as well as vasodilating properties and therefore reduces afterload; levosimendan; dobutamine; and intra-aortic balloon pump [29–31].

## Conclusions

We report a case of severe reversed stress cardiomyopathy in a patient with GBS, which required prompt management in terms of diagnosis and treatment. Stress cardiomyopathy is a rare complication during the acute phase of GBS and must be distinguished from autonomic dysfunction. Echocardiography is the gold standard for diagnosis, and acute coronary disease and myocarditis should be ruled out. Especially, ECG abnormalities, such as negative T waves, and hemodynamic instability in a patient with GBS should alert the clinician to the presence of stress cardiomyopathy. Because this diagnosis should not be missed, TTE should be systematically performed when repolarization abnormalities in ECG are present in GBS, even in asymptomatic patients. Early identification leads to an appropriate treatment strategy and improves prognosis.
